# The role of Vitamin D3 in ocular fibrosis and its therapeutic potential for the glaucomatous trabecular meshwork

**DOI:** 10.3389/fopht.2022.897118

**Published:** 2022-08-01

**Authors:** Alexander Morelli-Batters, Hannah C. Lamont, Mirna Elghobashy, Imran Masood, Lisa J. Hill

**Affiliations:** ^1^ School of Biomedical Sciences, Institute of Clinical Sciences, University of Birmingham, Birmingham, United Kingdom; ^2^ School of Chemical Engineering, Healthcare Technologies Institute, University of Birmingham, Birmingham, United Kingdom

**Keywords:** glaucoma, Vitamin D, POAG, trabecular meshwork, TGFβ, fibrosis

## Abstract

Glaucoma is the leading cause of irreversible blindness globally. The most prevalent subtype, Primary Open Angle Glaucoma (POAG), is characterized by increased intraocular pressure (IOP), damage to the optic nerve head and irreversible visual loss. IOP increases aqueous humor (AqH) outflow is reduced through the trabecular meshwork (TM) and Schlemm’s canal (SC). Increased outflow resistance is partly due to TM/SC dysregulation, including loss of normal trabecular meshwork cell (TMC) function, following increased levels of oxidative stress within TMC, dysregulated extracellular matrix (ECM) deposition and remodeling alongside alterations in TMC phenotype and apoptosis. Current widely available POAG treatments do not target the aberrant expression of ECM in the TM directly. As a result, most drug treatments can fail as the underlying pathological process continues unabated. Rho-kinase inhibitors have demonstrated the benefit of restoring TM/SC function, however there is a clear need to develop further treatment strategies that can target the underlying cellular processes which become dysregulated within the TMC during POAG pathogenesis. Vitamin D is suggested to be beneficial in alleviating the symptoms of fibrosis and inflammation in soft tissues. It has important functions in many major organ systems, including regulation of calcium, phosphate and parathyroid hormone. Evidence suggests that Vitamin D3 modulates ECM turnover through the conventional TGFβ-SMAD signaling, which is associated with the development of POAG. The link between Vitamin D3, inflammation and fibrosis within ocular tissues will be discussed and the potential roles of Vitamin D3 in the management of POAG patients will be explored within this review.

## Introduction

Aberrant extracellular matrix (ECM) turnover plays an important role in the pathogenesis of Primary Open Angle Glaucoma (POAG), the most prevalent form of glaucoma and a leading cause of irreversible blindness globally (1). In 2020, it was the cause of blindness in an estimated 3.6 million people over the age of fifty across the globe (1). POAG is characterized by a sustained increase in intraocular pressure (IOP) leading to irreversible damage to the optic nerve head and visual loss (2). The culprit for this dysregulation in IOP occurs in the anterior chamber of the eye, in two tissues: the trabecular meshwork (TM) and Schlemm’s canal (SC) (2). These two tissues mediate AqH outflow facility through constant remodeling of ECM, which then in turn regulates IOP (3, 4). Increased outflow resistance is due to TM/SC dysregulation, this is due to; loss of normal trabecular meshwork cell (TMC) function, increased levels of intracellular oxidative stress, upregulation of ECM protein expression and inflammatory markers, and subsequent densification of the TM ECM (2, 5, 6). ECM remodeling within the eye is intrinsically linked with many ocular pathologies, either as part of their pathophysiology or as a complication of disease; in the case of POAG, increased ECM protein expression and deposition within the TM is a key feature of its pathogenesis (7). However, the exact mechanisms of this pathogenesis are yet to be fully elucidated.

The lack of understanding surrounding the pathogenesis and the subsequent lack of effective treatments is responsible for many complexities in the management of POAG. Due to the limited number of agents that successfully attenuate the ECM changes seen in the TM/SC, such as Rho-kinase inhibitors, IOP lowering drops form the mainstay of current medical treatment (8). However these treatments, such as latanoprost and travoprost, do not target the underlying changes to the ECM within the TM/SC but instead reduce AqH production or attempt to increase outflow facility through the uveoscleral pathways (8). As a result, the efficacy of medical treatments is currently limited, with patients often requiring invasive surgical interventions (8). With current optimal treatment strategies, patients can still become blind, highlighting a clear unmet need to develop therapeutics that restore TM/SC function (8). It is believed that this could be achieved through targeting the dysregulated ECM turnover that occurs within the TM of patients with POAG. While the SC does not necessarily become dysfunctional through excessive ECM deposition, it has been noted in previous studies that TM and SC dysfunction are intimately linked (9).

It is becoming increasingly apparent that naturally occurring compounds may hold potential in targeting dysregulated ECM remodeling and fibrosis in several different organs (10–12). One such molecule is Vitamin D3, a critically important hormone with antioxidant and antifibrotic properties (13). It is well documented that levels of Vitamin D3 in the AqH and serum of patients with POAG can be significantly lower than that in control populations, and it is hypothesized that this deficiency may be responsible for ECM changes within the TM seen in the pathogenesis of POAG (14–16). In this review we will explore the application of Vitamin D3 as an antifibrotic agent in the ocular environment, more specifically, the TM.

## Dysregulated ECM remodeling in POAG

It is first important to understand the pathogenesis of POAG to appreciate the potential therapeutic target of fibrosis attenuation. The increased resistance in the TM that characterizes the condition has been attributed to increased tissue stiffness, dysfunctional ECM) remodeling and phenotypic changes, alongside widespread apoptosis of TMC (17, 18). This decrease in cellularity reduces the ability of the TM to adapt aqueous outflow facility in response to pressure changes within the eye. It has been noted that this the primary site of POAG pathogenesis occurs mainly in the juxtacanalicular (JCT) region of the TM (17). As tight regulation of ECM remodeling in the JCT is responsible for mediation of AqH outflow, excessive deposition occurs as a result of dysregulated protein expression in this region, disrupting homeostatic AqH outflow regulation (5, 18). TMC within the JCT express predominantly a fibroblast phenotype which is regulated through the TGFβ2 and SMAD 2/3 pathways, with overactivation causing pathological effects.

It has been suggested that a potential cause for the dysregulation of ECM remodeling is the TMC having the ability to undergo Type II Epithelial-Mesenchymal Transition (EMT) (19, 20). Type II EMT is a normal part of wound healing throughout the body, however its balance can shift in POAG, causing pathological changes associated with fibrosis (19). In TMC, this phenotypic transition involves a heightened transition state, from an epithelial state to a myofibroblast state, leading to expression of mesenchymal markers such as fibronectin and alpha Smooth Muscle Actin (αSMA) (19, 20). Myofibroblastic cells are commonly manifested during a fibrotic response, and therefore development of myofibroblastic-type TMC is thought to be responsible for the upregulation of ECM remodeling components (21–24). Moreover, excessive remodeling of the ECM within the TM leads to thickening of the elastic fiber sheaths and the accumulation of plaques, which are the most characteristic structural changes within the TM seen in POAG (25). It is these physical changes in tissue architecture that increase resistance to AqH outflow, and hence, IOP. A reduction in the remodeling of the ECM seen in POAG could therefore prevent the pathological increase in IOP, minimalizing any effect on AqH outflow facility. The involvement of fibrotic pathways in the pathological changes to the TM seen in POAG provides potential to modulate the pathology of POAG harnessing the anti-fibrotic potential of treatments, such as Vitamin D3.

## Vitamin D3 and dysregulated ECM remodeling in the TM

Vitamin D refers to a group of fat soluble secosteroids that perform an endocrine function through Vitamin D receptor (VDR) modulation (13). It has several well documented roles in the human body, most notably calcium absorption and regulation, influencing metabolic disease, muscle strength, immune and inflammatory systems, while exhibiting antioxidant properties (13, 26–29). Associations between Vitamin D3 and fibrosis are increasingly being identified, leading to postulation that it may have valuable potential as an antifibrotic (30–36).

Vitamin D3 has been shown to act through ocular VDRs in several ocular pathologies. Stimulation of the VDR in respective tissues results in reduced inflammation in dry eye disease, age-related macular degeneration (AMD), inhibition of retinal neovascularization, protection of RPE cells from oxidative damage and reduction of corneal wound scarring (37–46). This evidence suggests that Vitamin D3 may also have the potential to modulate the VDR in the TM in order to attenuate excessive ECM deposition in the TM (47).

To further study the use of Vitamin D3 in POAG, it is vital to understand the effects of VDR stimulation in the TM; most crucially, its ability to modulate the pathways responsible for the pathological changes that affect the TM in POAG. Since the discovery of elevated concentrations of TGFβ2 in the eyes of POAG patients in 1994, this profibrotic messenger has been widely considered to be the key driving factor in the pathology of the TM seen in POAG (48, 49). It is postulated that the upregulation of TGFβ2 observed in POAG could be due to conditions of oxidative stress (50–52). TGFβ2 is a cytokine with several isoforms that regulates differentiation, motility and organization of myofibroblast-like cells and is necessary for the normal regulation of TMC for homeostatic AqH outflow (47). It plays a key role in wound healing and altering the production of fibronectin and collagen. Dysregulation within these systems can lead to major pathological changes, and thus it is credited as one of the major pro-fibrotic pathways (48, 50).

Almost all cells in the body have receptors for TGFβ, and TMC are no different (23, 53, 54). Indeed, TGFβ2 has been implicated in the ECM changes seen in the TM of POAG patients. Studies have shown that TGFβ2 can increase ECM remodeling in the TM by antagonizing the activity of matrix metalloproteinases (MMP) through upregulating the MMP inhibitor PAI-1, and to upregulate the expression of tissue transglutamase (TTG) (24, 55). MMPs are responsible for the breakdown of ECM and TTG is a protein that can cross-link fibronectin to increase ECM formation (24, 55). TGFβ2 has also been shown to induce oxidative stress in TM cells, a potential explanation for increased apoptosis (51). Furthermore, TGFβ has been observed to drive the EMT process within the TM of patients with POAG (56, 57). It has also been found that areas of low AqH outflow facility within the TM correspond to areas of high TGFβ2 expression, and likewise TGFβ2 administration in *ex vivo* studies have been shown to increase IOP alongside decreased AqH outflow (58–61). There are many additional cytokines and genes implicated in the pathological changes to the TM seen in POAG, including: CDKN2B-AS1 and CDKN2B genes, IL-1, IL-6, TNF-α, VEGF and FGF (62, 63). Nevertheless, many exert their actions through TGFβ2 signaling, further supporting the role of TGFβ2 as the main driving force in the pathogenesis of POAG (62–64). In this way, the ability of Vitamin D3 to modulate the action of TGFβ2 is vital to its potential therapeutic value in POAG.

**Figure 1:**
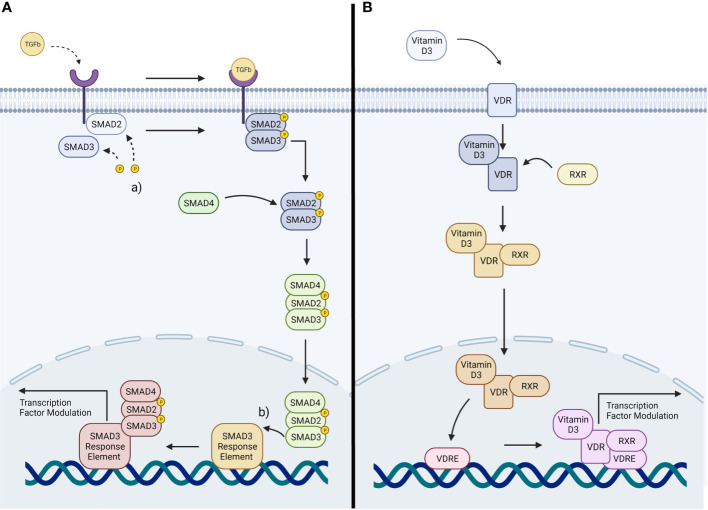
**(A)** Representation of TGFβ signaling: TGFβ cytokines activate TGFβ receptors, activating the SMAD2/3 pathway. This induces a transcriptional change within the nucleus. (a) phosphorylation of SMAD2/3. (b) binding of the SMAD complex to its response element **(B)** Representation of Vitamin D3 signaling: Vitamin D3 stimulates the VDR, activating a downstream signaling pathway resulting in transcriptional change within the nucleus.

TGFβ2 acts through several known signaling pathways to drive cellular changes, with conventional TGFβ2 cascades referred to as the SMAD pathways (56). These contain constituent intracellular SMAD signaling proteins associated with regulation of corneal scarring and ocular immune privilege (65, 66). The unconventional cascades include MAPK and Rho GTPase pathways (67, 68). In the conventional pathways, TGFβ first binds to its cognate TGFβ receptors (TGFβRI and TGFβRII) (69). This causes a cascade of intracellular events, culminating in phosphorylation of cytosolic protein effectors, the SMAD-2/3 proteins (70). Activated SMAD proteins form a complex with SMAD-4, the SMAD carrier, by which they are transported to the nucleus (69). Once inside the nucleus the complexes are able to recruit various coactivators and corepressors in order to induce transcriptional change (**Figure 1A**) (71). These expressional changes include upregulation of fibronectin, aSMA and collagens (72). The conventional SMAD-2/3 pathway has specifically has been implicated in the development of fibrosis within other soft tissues, with studies showing that SMAD3 negative knockout mice are resilient to the induction of intestinal fibrosis (73).

The proposed mechanism for the interaction between Vitamin D3 and the attenuation of fibrosis involves modulation of the same TGFβ pathways involved in the pathogenesis of POAG, through the stimulation of the VDR (66).

There are parallels between TGFβ signaling and VDR signaling. In the presence of Vitamin D3 the stimulation of the VDR leads to modulation of gene expression within a cell (74). Vitamin D3 binds with high affinity to VDR (75). This leads the VDR to translocate to the cell nucleus, forming a heterodimer with the retinoid X receptor (RXR) (76). The resultant Vitamin D3 Receptor-Retinoid X Receptor heterodimer (VDR-RXR) then binds to Vitamin D3 response elements (VDRE) on the DNA of the cell (76) (**Figure 1B**). The heterodimer has the propensity to interact and bind with a large number of transcription factors, allowing modulation of the expression of many genes (77). Due to the nature of this mechanism, it is thought that the VDRE must share the same chromosomal domain as the genes it is to modulate (78, 79). It is also thought that the ability for VDR signaling to impact TGFβ signaling is due to the close proximity of their cistromes; as the chromatin binding sites for the VDR and SMAD3 are within a nucleosome distance (66, 78, 79).

**Figure 2:**
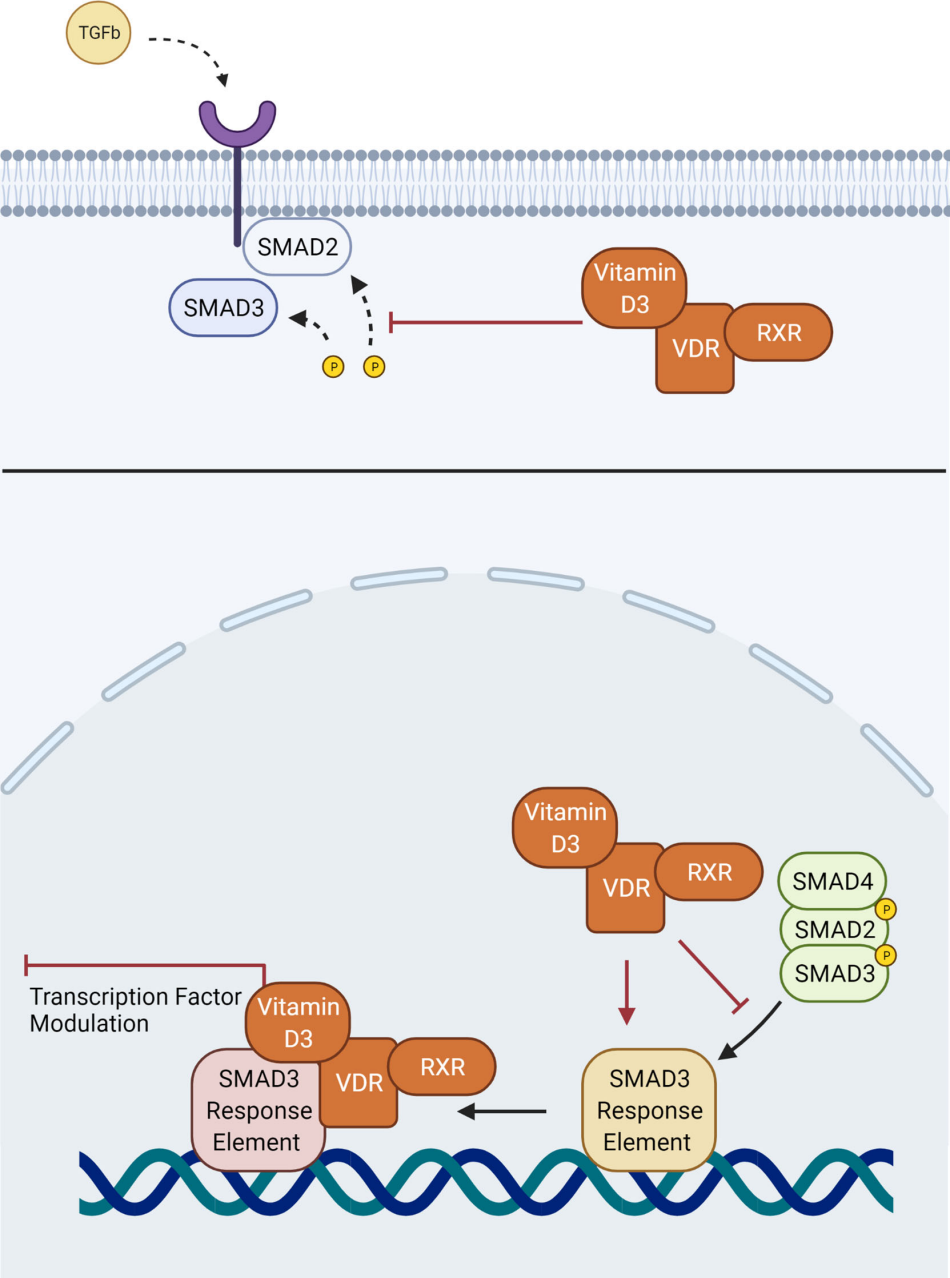
Interaction of the VDR-RXR heterodimer with aspects of the conventional TGFβ SMAD signaling pathway. Vitamin D3 may interfere with phosphorylation of SMAD2, or binding of the downstream SMAD complex with its response element.

The proximity of these two pathways means that they have the potential to interact in several ways within myofibroblast-like cells such as the TMC to modulate phenotype. Previous studies assessing hepatic stellate cells, another myofibroblast-like cell type, have suggested that the VDR-RXR heterodimer is a competitive antagonist to SMAD3 binding of chromatin at the SMAD3 domain (66). By blocking SMAD3 binding, Vitamin D3-mediated stimulation of the VDR prevents transcriptional upregulation of profibrotic factors, such as fibronectin, α-SMA and collagen (**Figure 2**) (66). Experimentation on hepatic stellate cells has also offered an alternative hypothesis on the interaction between TGFβ2 and the VDR. The VDR-RXR complex is known to bind a large number of transcription factors, leading to the suggestion that rather than binding to the SMAD3 binding site directly, it binds to shared transcription factors (71). This in turn inhibits the ability of SMAD3 to induce transcriptional change, acting to antagonize stimulation of a fibrotic response by TGFβ2 (71). It has been further reported that in skin fibroblasts of patients with systemic sclerosis, that competitive binding of the VDR to the SMAD3 cistrome prevented profibrotic transcription (**Figure 2**) (80). Alternatively, in cardiac fibroblasts and hepatic stellate cells the mechanism appeared to involve inhibition of SMAD2 phosphorylation (81, 82).

Regardless of which aspects of the downstream signaling pathways interact between these different myofibroblastic-type cells, previous evidence shows that Vitamin D3 is able to act through VDR mediated downstream signaling to downregulate TGFβ induced SMAD transcriptional action. It is reasonable to suggest that Vitamin D3 could also act through the VDRs of myofibroblastic-type TMC to modulate the conventional TGFβ pathways involved in ECM remodeling. To add weight to this argument, polymorphisms within the VDR have been identified (Cdx-2, Fok-1, Bsm-1 and Taq-1) as genetic risk factors for the development of POAG, reinforcing the relationship between Vitamin D3 and pathogenesis of POAG (83).

In light of TGFβ2 being both a pathogenic driver in POAG, and the pathway through which Vitamin D3 attenuates fibrosis, a recent study investigated the effect of Vitamin D3 on the conventional TGFβ pathways in TMC (47). Oxidative stress was induced in TMCs using hydrogen peroxide in order to upregulate TGFβ and SMAD3 expression (47). Subsequent administration of Vitamin D3 reduced ECM modelling when compared to controls alongside down-regulation of TGFβ and SMAD3 (47). This provides encouraging evidence that Vitamin D3 is able to modulate the TGFβ pathway to attenuate ECM remodeling in the TM. Future studies could administer TGFβ directly to TMC, rather than increasing TGFβ expression indirectly. This would enable more direct conclusions to be drawn without confounding from other variables resulting from hydrogen peroxide administration. Vitamin D3 was also found by this study to reduce oxidative damage in TMC (47). This was again attributed to Vitamin D3 reducing activation of the TGFβ pathways in the presence of oxidative stress (47). Vitamin D3 has also been shown to minimize oxidative damage in corneal epithelial cells, retinal pigment epithelium and cone cells (37, 38, 84). In this way, Vitamin D3 may not only prevent TGFβ action, but also mechanisms of TGFβ activation.

It is important to assess whether the regulation of TGFβ action and ECM deposition within the TM achieved by Vitamin D3 can influence IOP and optic neuropathy in *in vivo* models. A recent paper showed that interference in TGFβ pathways, albeit not by Vitamin D3, translated to an increase in flow of AqH through the TM and a reduction in IOP in rabbit models (85). This suggests that interrupting TGFβ2 signaling within the TM does have beneficial effects on lowering IOP. By doing so, it has also been shown that the resultant reduction in IOP has the desired effect on the neuropathy at the heart of POAG; a study reported that a decrease in TM fibrosis and subsequent reductions in IOP were responsible for a reduced loss of retinal ganglion cells in a glaucomatous rodent model (86). Such evidence, overall, provides compelling encouragement for the effectiveness of reducing ECM deposition within the TM through Vitamin D3 therapy in POAG as a way to reduce IOP and glaucomatous optic neuropathy (87).

## Discussion - therapeutic value and future research

This review has discussed the notion that Vitamin D3 has the potential to modulate the TGFβ signaling pathway responsible for the pathological ECM remodeling that occurs to the TM in POAG. To add weight to this hypothesis, Vitamin D3 therapies have also been shown to potentially lower IOP and reduce inflammatory responses in retinal ganglion cells in rodent models regardless of its antifibrotic effect in the TM (88, 89). This suggests that should Vitamin D3 have other beneficial effects on cellular dysfunctionality, asides from ECM remodeling alterations, it may still provide therapeutic benefit in the management of POAG.

A proportion of patients with POAG require surgical treatment regardless of treatment strategy (90). This is largely due to patients becoming refractory to existing treatments; with Vitamin D3 operating through alternative mechanisms to existing therapies, it could pose as an additional treatment to be undertaken prior to surgery (90). This would increase the time before which each patient was required to undergo surgery and hazard its risks. Vitamin D3 may also play a beneficial role in the outcome of these surgeries themselves (90–92). An increase in TGFβ has been linked to complications of glaucoma surgery such as corneal scarring and conjunctival fibrosis which occur due to tissue insult; it has been shown that modulation of TGFβ action through the use of an alternative agent greatly reduced conjunctival fibrosis (90–92). Through modulation of the same pathways, future research may provide evidence that Vitamin D3 can also improve surgical outcomes.

While current research shows a promising path for Vitamin D3 as an antifibrotic in several different soft tissues, it is important to also note the potential limitations of Vitamin D3 as a TGFβ modulator in POAG (31, 33, 80, 81). Current research provides evidence for the action of Vitamin D3 on conventional TGFβ pathways, however future research should assess the possibility that SMAD independent pathways could be upregulated to bypass the action of Vitamin D3 through increased expression of MAPK and Rho kinases. In addition to this, despite promising findings in rodent models, it has been debated that Vitamin D3 levels had no significant association with IOP in a non-glaucomatous human cohort (93) but the potential of Vitamin D3’s benefits in glaucomatous patients should be further explored.

## Conclusion

This review has explored the association between Vitamin D3 and ocular fibrosis pathways, with the aim of assessing its viability as a novel therapeutic in the treatment of POAG. It represents a promising agent in reduction of ECM deposition in the TM through its modulation of the TGFβ signaling pathway associated with the development of a fibrotic response associated with POAG. Vitamin D3 has been shown to antagonize TGFß; signaling and promote antifibrotic change within myofibroblastic-type cells, akin to the TMC of patients with POAG. Further research on the implications of reducing ECM deposition in animal models emulating POAG is needed to draw firm conclusions on the effectiveness of Vitamin D3 in attenuating these changes and lowering IOP.

## Author contributions

AM-B and HL contributed equally to preparation of draft and review of the manuscript. ME, IM, and LH edited and reviewed the manuscript. All authors contributed to the article and approved the submitted version.

## Funding

This work was supported by EPSRC and SFI Centre for Doctoral Training in Engineered Tissues for Discovery, Industry and Medicine (Grant number EP/S02347X/1).

## Acknowledgments

All illustrations were created with Biorender.com (accessed 13/03/2022)

## Conflict of interest

The authors declare that the research was conducted in the absence of any commercial or financial relationships that could be construed as a potential conflict of interest.

## Publisher’s note

All claims expressed in this article are solely those of the authors and do not necessarily represent those of their affiliated organizations, or those of the publisher, the editors and the reviewers. Any product that may be evaluated in this article, or claim that may be made by its manufacturer, is not guaranteed or endorsed by the publisher.
